# Look out for the Pendulum

**Published:** 1913-06-15

**Authors:** C. N. Johnson


### LOOK OUT FOR THE PENDULUM.

BY C. N. JOHNSON, M.A., D.D.S.

The profession seems to run to fads and fancies, in fact all professions do. A man of some authority makes a statement and the lambs follow where the bellwether jumps. In the early history of the profession too many teeth were extracted. It was not unusual for a practitioner to condemn a tooth if it became abscessed, and so numberless teeth were needlessly sacrificed and people were deprived of useful organs which were essential to their highest efficiency. This fact became apparent to the thinking men of the profession and they instituted a reform whereby the natural teeth were to be saved by treatment and proper filling. It became almost a disgrace to extract a tooth, and every effort was made to save them. Great good followed this propaganda and the people themselves were educated to appreciate the value of a natural tooth and the possibility of saving it even after it had become affected by disease. All sorts of medicaments and technical procedures were brought out to cure abscessed teeth, and the slogan went up that no tooth should be sacrificed that could not be extracted with the fingers. Article after article was published also on the treatment of teeth affected by pyorrhea, and there was the most laudable attempt to prevent people from suffering the necessity of wearing artificial dentures.

This was a move in the right direction, but like many other praiseworthy efforts it was carried too far. All teeth could not be cured of abscess or pyorrhea, and many of them were nursed along for years with puss discharging into the mouth in the vain attempt to eventually cure them and in blind subjugation to the dogma that natural teeth should not be extracted till they became hopelessly loose. Little cognizance was taken of the possible ill effects of pus being re-absorbed into the system causing constitutional affections. Recently there has been an awakening and reversal of former ideas, and now look out for the pendulum. It is likely to swing back to the dangerous days of too free extraction. Papers have been read and fully discussed on the evils of systemic infection from abscessed and pyorrhea teeth. Men are holding high their hands in horror at the "sepsis traps" of artificial crowns and bridges, and woe betide the man who has the assurance to stand up and say that there are sanitary crowns and sanitary bridges. But there are. True as it is that many teeth have been retained in the mouth when the best interests of the patient would have been subserved by their extraction, and also true that there have been many crowns and bridges that were a menace to the patient's health; but the fact still remains that most diseased teeth, can be made healthy by proper treatment, and that crowns and bridges can be so constructed as to be cleanly and advantageous to the patient. Let us not recede from the high ideals of our immediate predecessors who valued a natural tooth beyond the estimate of money, and who contended for its salvation to the last extremity. By all means let us learn that it is not safe to leave pus pockets in the mouth; eliminate the pus even at the expense of losing the tooth, but first stand by the conviction that it is seldom necessary to lose the tooth in order to control the pus.

A short time ago a prominent medical man read a paper in which he traced rheumatic affections in some cases to diseased tonsils. Instantly all over the land tonsils began to be slaughtered by the wholesale—innocent and guilty alike they fell before the irresistible sweep of a passing craze. Before the pendulum swung back to norman the land in certain sections was strewn with tonsils, some of which would have served a better purpose in the economy of life if they had been allowed to remain with the patient. The same thing will hold true of the teeth unless the good sense of the dental profession prevents it from stampeding at a critical moment. The old adage is a safe rule to follow: "Prove all things; hold fast to that which is good."—*Dental Review*, May 1913—p. 452.

